# Sequence Polymorphism in Candidate Genes for Differences in Winter Plumage between Scottish and Scandinavian Willow Grouse (*Lagopus lagopus*)

**DOI:** 10.1371/journal.pone.0010334

**Published:** 2010-04-23

**Authors:** Pontus Skoglund, Jacob Höglund

**Affiliations:** Department of Population Biology and Conservation Biology, Uppsala University, Uppsala, Sweden; Centre National de la Recherche Scientifique, France

## Abstract

**Background:**

Population variation in the degree of seasonal polymorphism is rare in birds, and the genetic basis of this phenomenon remains largely undescribed. Both sexes of Scandinavian and Scottish Willow grouse (*Lagopus lagopus*) display marked differences in their winter phenotypes, with Scottish grouse retaining a pigmented plumage year-round and Scandinavian Willow grouse molting to a white morph during winter. A widely studied pathway implicated in vertebrate pigmentation is the melanin system, for which functional variation has been characterised in many taxa.

**Methodology/Principal Findings:**

We sequenced coding regions from four genes involved in melanin pigmentation (*DCT*, *MC1R*, *TYR* and *TYRP1*), and an additional control involved in the melanocortin pathway (*AGRP*), to investigate the genetic basis of winter plumage in *Lagopus*. Despite the well documented role of the melanin system in animal coloration, we found no plumage-associated polymorphism or evidence for selection in a total of ∼2.6 kb analysed sequence.

**Conclusions/Significance:**

Our results indicate that the genetic basis of alternating between pigmented and unpigmented seasonal phenotypes is more likely explained by regulatory changes controlling the expression of these or other loci in the physiological pathway leading to pigmentation.

## Introduction

Studies on genetic variation in endangered species have traditionally focused on neutral genetic markers such as microsatellites or mtDNA, but the importance of functional genetic variation is receiving increased interest. However, characterising ecologically significant functional variation in wild species for which no closely related model organism exists remains difficult, and is further complicated in species which are subject to active conservation programs due to restrictions against invasive sampling. A plausible non-invasive approach to study adaptive variation in threatened species is to utilize candidate genes for interesting phenotypes that are known a priori or from mapping experiments in related model species [1]. One such phenotype is geographic variation in seasonal polymorphism, exemplified by the white winter phenotype displayed by many arctic animal species. While such seasonal phenotypic variation might be expected to have a complex genetic basis, it has been recently shown that non-synonymous mutations in a candidate gene perfectly explain population variation in winter coat colour among Arctic foxes (*Alopex lagopus*) [2].

Colouration stands as perhaps the most extensively studied and described phenotype in natural animal populations. Two adaptive hypotheses of phenotypic variation that have been critically tested in an evolutionary framework are camouflage e.g. [3] and sexual colouration [4]. These and most studies on animal pigmentation to date have focused on the melanin system, in which specialized cells known as melanocytes produce either of two pigments—eumelanin or pheomelanin—resulting in different tone and colour of skin, hair and feathers. High concentration of eumelanin leads to a dark appearance, whereas pheomelanin usually results in a brown or reddish phenotype. Several genes that are involved in the pathway have been identified in mammals (reviewed by [5]), of which the melanocortin 1 receptor gene (*MC1R*) is the most well known.

The Willow grouse (*Lagopus lagopus*) and its subspecies the Scottish grouse (*Lagopus lagopus scoticus*) display marked seasonal differences in their plumage. While Scandinavian Willow grouse, like a few other vertebrate arctic and subarctic species, are predominantly white all through winter, they moult to a pigmented plumage during the snow-free season. In contrast, the Scottish grouse which inhabit the British Isles with a warmer climate retain their pigmented plumage all through the year. Intuitively, one would expect that the cryptic function of the pigmented vs. white phenotype is strongly related to snow coverage—leading to differences in individual fitness in the respective environments.

Previous studies have identified several causative non-synonymous substitutions in avian *MC1R* exons, including *Val85Met* [6], [7], *Glu92Lys* [8], *Arg230His* [9] and others [10], [11]. Moreover, the evolutionary rate of non-synonymous substitutions has been shown to be correlated with sexual dimorphism in Galliform birds [4]. Several alleles that contribute to pigmentation have been described in *MC1R* orthologs in other vertebrates, most notably *Arg65Thr* [12]. In *TYRP1*, Nadeau and colleagues [13] described a *Phe282Ser* mutation that was associated with a plumage phenotype in Japanese quail (*Coturnix japonica*). Conversely, phenotypic variation in plumage was not related to *MC1R* variation in the blue crowned manakin, *Lepidothrix coronata* [14] or old world leaf warblers, *Phylloscopus sp*. [15], but these studies did not investigate other candidate genes.

We sought to investigate any associations between phenotypic differences in winter plumage and coding region variation by obtaining sequences from four previously identified candidate genes for plumage coloration (*MC1R, TYR, TYRP1* and *DCT*) in European *Lagopus* populations. A fifth additional gene (*AGRP*) is implicated in the melanocortin pathway but not pigmentation and was included mainly as a control [4], Mutations in Tyrosinase (*TYR*), Tyrosinase-related protein-1 (*TYRP1*), and DOPA-chrome tautomerase (*DCT*, also known as Tyrosine-related protein-2) are mostly known for conferring phenotypic changes along the yellow-brown axis, and the three genes all belong to the tyrosinase family. On the other hand, the melanocortin 1-receptor (*MC1R*) is expected to determine phenotypic change from dark pigmentation to white.

## Results

### PCR amplification and sequencing

We obtained exonic and intronic sequences from *AGRP*, *DCT*, *MC1R*, *TYR* and *TYRP1* spanning chicken nucleotide positions 25–536, 309–587, 104–900, 68–375, 7–819 and 68–375, respectively. Due to varying amplification success, the number of successfully sequenced individuals varied between 7–13 from Scandinavian Willow grouse and 7–11 from Scottish Red grouse.

### Sequence variation

**Figure 1:**
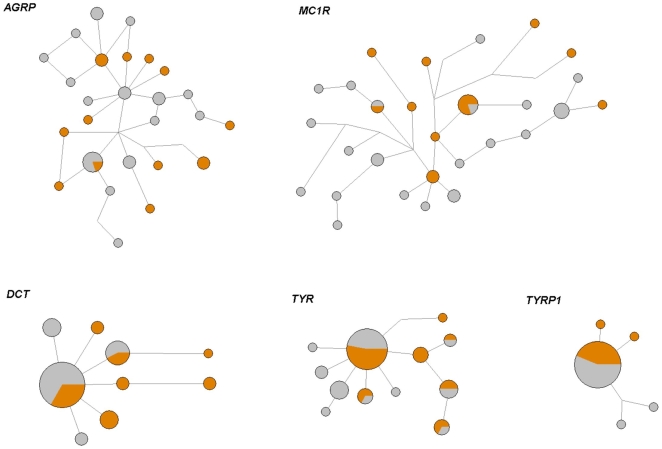
Median-joining haplotype networks of phased sequences. Scandinavian Willow grouse and Scottish Red grouse sequences are represented with grey and red, respectively. Node size is proportional to haplotype frequency.

In the final alignments spanning a total of 2626 bp, we observed 65 segregating sites, corresponding to 1 SNP per ∼40 bp, which is in concordance with previously published levels of exonic polymorphism in the genus [16]. None of the found alleles separated the two phenotypically distinct subspecies (Fig. 1). For instance, sequence variation in *MC1R* did not show any correlations with colour in a region spanning amino-acid position 36 to 300. Moreover, several shared polymorphisms were found between the populations, and this was reflected in the level of population differentiation (F_ST_<0.10; Table 1). Nucleotide diversity corresponded to previous estimates of *π* = 10^−3^ in birds and other loci from the studied *Lagopus* populations [16].

**Table 1:** Summary statistics of genetic variation and statistical tests of neutrality for phased sequence data used in the study.

	*n*	*S*	*π*	*θ_W_*	*D*	*H*	F_ST_
*DCT*	48	5	0.0020	0.0049	−1.40	0.45	0.087
Scandinavia	26	1	0.0006	0.0011	−0.71	0.14	
Scotland	22	4	0.0036	0.0048	−0.70	0.69	
*MC1R*	38	22	0.0057	0.0047	−1.22	0.07	0.097
Scandinavia	24	15	0.0057	0.0069	−0.61	−0.21	
Scotland	14	12	0.0044	0.0056	−0.85	1.31	
*TYR*	46	11	0.0025	0.0035	−0.81	0.51	0.015
Scandinavia	24	10	0.0025	0.0038	−1.09	0.29	
Scotland	22	6	0.0024	0.0022	0.27	0.54	
*TYRP1*	36	4	0.0012	0.0045	−1.89*	0.32	0.024
Scandinavia	20	2	0.0014	0.0032	−1.45	0.35	
Scotland	16	2	0.0009	0.0022	−1.49	0.23	
*AGRP*	36	23	0.0062	0.0095	−1.18	0.39	0.025
Scandinavia	22	10	0.0052	0.0051	−0.07	−0.33	
Scotland	14	21	0.0081	0.0112	−1.19	0.88	

Statistical significance is indicated with a * (P<0.05). All results are given for the whole dataset as well as the Scandinavian and Scottish subpopulations: *n* is the number of (phased) sequences, *S* is the number of segregating sites, *π* is the pairwise nucleotide diversity, *θ_W_* is Watterson's estimator of the population mutation rate given by *S*, *D* is Tajima's test of departure from the standard neutral model using the folded frequency spectrum, *H* is Fay and Wu's test of departure from the standard neutral model using the unfolded frequency spectrum, F_ST_ is Wright's fixation index.

### Statistical tests of selection

Tajima's *D* statistic [17] was generally negative across all loci (Table 1), also compatible with the findings of [16]. Statistical significance for deviation from the standard neutral model was only obtained from *TYRP1*, but since this is based on 4 SNPs in just 267 bp, we are careful in interpreting this result as non-neutral evolution of the locus. Tajima's *D* is the normalized difference between nucleotide diversity (*π*) and Watterson's estimator of *θ* and tends to negative when there is an excess of low frequency variants in the sample, and positive when there is an overrepresentation of intermediate frequency variants. In the case of a recent selective sweep, Tajima's *D* is expected to take a negative value more extreme than that of the genomic background. However, since Tajima's *D* does not distinguish which allele is ancestral, it only considers the folded frequency spectrum. In contrast, Fay and Wu's *H* statistic [18] can identify an excess of derived alleles in a sample by using an outgroup sequence. To this end we calculated *H* across the whole sequence (Table 1) and in a sliding window of 100 bp with 25 bp steps (data not shown), but did not find significant evidence of a recent selective sweep in the history of either subspecies. However, we note that *MC1R* displays the most extreme values of *H* in the two populations, with a negative value for the Scandinavian population and a positive value for the Scottish population (Table 1).

### Linkage disequilibrium

We also investigated linkage disequilibrium since the extent of correlations between markers determine the ability to detect selection acting on nearby regions. In line with previous results [16], we found low and homogenous levels of linkage disequilibrium in the three loci with >500 bp sequence data (*AGRP1*, *MC1R* and *TYR*), with ZnS [19] values of 0.06 in all cases.

## Discussion

Arctic species which display a white non-melanized winter coat or plumage include hares, grouse, wolves, mustelids, owls and foxes. Våge and coworkers [2] showed that a rare blue phenotype in arctic foxes in Scandinavia was perfectly associated with two amino acid substitutions in *mc1r*. Presently, northern European Arctic foxes are highly endangered and threatened by global climate change. A recent study showed that after the last glaciation, arctic fox populations were not able to track their optimal habitat when the climate shifted [20]. If this is also the case in present populations of other arctic species, genetic diversity that preserves adaptability to changing climate conditions might be paramount for their long-term survival. The willow/red grouse is an example of a polymorphism that is associated with climatic conditions, with red grouse being cryptic all year round while willow grouse have a camouflaged all-white winter plumage. It could be hypothesised that the non-white morph will be favoured when the climate becomes warmer and winters become snow-free. However, the non-white populations in Britain and Ireland are presently not in contact with the closest white populations in Scandinavia, being separated by the North Sea, and thus it is unlikely that the non-white morph will expand via migration. Instead Scandinavian willow grouse must respond via selection favouring a non-white mutant arising within the Scandinavian population. This requires genetic variation for, or possibilities to mutate to, the non-white phenotype in the Scandinavian population. Interestingly, local populations living on largely snow free islands off the Norwegian coast show signs of less pronounced white winter plumages (J.H. pers. obs.). Understanding the genetic basis of this polymorphism and whether similar genetic changes can be found in all arctic species is thus of great importance.

We did not observe any non-synonymous substitutions associated with the differing winter plumage phenotypes in Willow grouse in the sequenced regions of *AGRP1*, *DCT*, *MC1R*, *TYR* or *TYRP1*. Additionally, we did not detect any significant deviations from the standard neutral model which could indicate recent selection in regions closely linked to the ones studied. However, we note that because of the rapid decay of linkage disequilibrium and short haplotype blocks in this species recently reported by [16] and confirmed in our data, a selective sweep affecting *cis*-regulatory elements or exonic regions that were not covered by our data could still be present on a relatively short physical distance from the sampled loci. In fact, we did observe a non-significant excess of high-frequency derived alleles in the *MC1R* sequences of the Scottish population, a pattern that would be compatible with a recent sweep in the vicinity of the exon. Unfortunately, while this could be resolved by characterising population variation in the regions close to the exon, this remains very difficult since high intergenic variability prevents repeatable PCR amplification. However, a role for any of the five loci in maintaining the marked phenotypic differences is discouraged by the complete lack of lineage sorting into the two geographic populations. Willow grouse and Scottish red grouse populations are genetically differentiated [16], so a hypothetical locus that governs or contributes to the phenotypic diversity which is the basis of their taxonomic classification is expected to have a phylogenetic signature that mirrors the origin of the sampled sequences.

Over 100 loci are believed to affect pigmentation in vertebrates [21], and while the discovery of convergent evolution of genetic background to similar phenotypes between quite disparate taxa such as rock pocket mice (*Chaetodipus intermedius*) [22] and Arctic skuas [9] encourages further surveys of *MC1R*, this study shows that attention needs to be directed to other parts of the genome as well. As mentioned above we can not exclude changes in regulatory genes affecting the sequenced loci but we were unable to find any changes associated with the colour polymorphism within any of the sequenced genes. The nature of any putative regulatory loci is highly speculative, but we would like to draw attention to the possibility that the polymorphism might be driven by changes in the moult pattern. Red grouse have two annual plumages and associated moults while willow grouse have three [23]. Having lost one plumage (the third all white winter plumage) has allowed the red grouse to adapt to the snow free winter conditions on Britain and Ireland. Thus candidate loci that are involved in circadian rhythms and putatively in the timing and onset of moult, like *Clock*-genes [24], might be suitable candidate loci in the quest for the genetic basis of this adaptation.

## Materials and Methods

### Ethics statement

Samples from Scandinavia were all obtained from shot birds which were culled under local laws and guidance in the respective region of origin. Samples from Scotland came from birds caught and bled under government license to Aberdeen University. These birds were released after processing. Animal research at Uppsala University follows the guidelines provide by Swedish national legislation (http://www.codex.uu.se/).

### PCR amplification and sequencing

Genomic DNA was extracted from 13 Willow grouse (*Lagopus lagopus*) from Tjallingbacken, Sweden, and 15 Red grouse from Glas Choille, Scotland, United Kingdom, using salt-based precipitation as in [16]. Primers for Galliform loci originally described by [4] were screened for functionality in European *Lagopus* and five primer pairs were chosen (AGRPF1-AGRPR7, DCTF2-DCTR1, MSHR80-MSHR9, TYR1F-TYR1R, TP1e1F3-TP1e1R1). Polymerase chain reactions (PCR) were run in 20 µl volumes with 2.5 mM dNTP, 2.5 mM MgCl_2_, 1x reaction buffer (Fermentas, Vilnius, Lithuania), 1 mM of each primer, 0.5 units of Taq polymerase (Fermentas) and 30–70 ng DNA. The cycling parameters included an initial denaturation step of 94°C for 2 min followed by 35 cycles of 94°C for 30 s, 60–65°C for 45 seconds and 72°C for 1 min and a final elongation of 72°C for 2 min. Annealing temperatures were 65°C for the *MC1R* locus, 63°C for *TYR* and 60°C for *TYRP1*, *AGRP* and *DCT*. PCR products were sequenced directly using the amplification primers using Big Dye v3.1 on a 3730xl automated sequencer (Applied Biosystems, Foster City, CA, USA) by a commercial sequencing service (Macrogen, Seoul, Korea) and a MegaBace 1000 capillary instrument (GE Healthcare, Wakeusha, Wisconsin, USA) using Dyenamic ET terminators (GE Healthcare) according to the manufacturer's instructions.

### Sequence analysis

Sequences were assembled and manually edited with CodonCode aligner (CodonCode Corporation, Deadham, MA, USA) and sequence alignments were created with MUSCLE using default parameters [25]. Reading frame was inferred by comparing with the chicken (*Gallus gallus*) genome sequence [26] and all alignments were manually inspected for non-synonymous and synonymous polymorphisms that clustered within either Scottish or Scandinavian populations. Haplotypes were inferred using PHASE [27] with 1000 iterations and a 100 generation burnin (Sequence File S1). Summary statistics for genetic polymorphisms, population differentiation and linkage disequilibrium as well as statistical tests of neutrality were calculated with DnaSP [28]. Variation was surveyed across the whole sequences, totaling over synonymous and non-synonymous sites. Wright's fixation index F_ST_ [29] was calculated as in [30]. Linkage disequilibrium was examined with the ZnS statistic [19], which is the average of r^2^ [31] over all polymorphic markers. Fay and Wu's *H* test [18] was performed by determining the derived allele from comparisons with black grouse (*Tetrao tetrix*) sequences described by [4], and statistically significant deviations from the standard neutral model were investigated using coalescent simulations based on the observed population mutation rate. To further illustrate differentiation and reveal stratification within the candidate genes, median-joining networks [32] of phased haplotypes were constructed using Network 4.510 (http://www.fluxus-engineering.com/).

## Supporting Information

Sequence File S1 Phased sequences.
(0.00 MB ZIP)
